# Over prescribing of antibiotics for acute respiratory tract infections; a qualitative study to explore Irish general practitioners’ perspectives

**DOI:** 10.1186/s12875-019-0917-8

**Published:** 2019-02-14

**Authors:** Jane O’Doherty, Leonard F. W. Leader, Andrew O’Regan, Colum Dunne, Soorej Jose Puthoopparambil, Raymond O’Connor

**Affiliations:** 1 Graduate Entry Medical School, University of Limerick, Limerick, Ireland; 2 Royal College of Surgeons in Ireland - Medical University of Bahrain, Busaiteen, Muharraq Governorate, Kingdom of Bahrain; 3 Centre for Infections in Infection, Inflammation & Immunity (41), Graduate Entry Medical School, University of Limerick, Limerick, Ireland; 4 International Maternal and Child Health (IMCH), Department of Women’s and Children’s Health, Uppsala University, Uppsala, Sweden

**Keywords:** Antibiotic resistance, Antibiotics, General practice, Acute respiratory tract infections

## Abstract

**Background:**

Anti-microbial resistance (AMR) is a global threat to public health and antibiotics are often unnecessarily prescribed for acute respiratory tract infections (ARTIs) in general practice. We aimed to investigate why general practitioners (GPs) continue to prescribe antibiotics for ARTIs despite increasing knowledge of their poor efficacy and worsening antimicrobial resistance.

**Methods:**

We used an explorative qualitative study design. Thirteen GPs were recruited through purposive sampling to represent urban and rural settings and years of experience. They were based in general practices within the Mid-West of Ireland. GPs took part in semi-structured interviews that were digitally audio recorded and transcribed.

**Results:**

Three main themes and three subthemes were identified. Themes include (1) non-comprehensive guidelines; how guideline adherence can be difficult, (2) GPs under pressure; pressures to prescribe from patients and perceived patient expectations and (3) Unnecessary prescribing; how to address it and the potential of public interventions to reduce it.

**Conclusions:**

GPs acknowledge their failure to implement guidelines because they feel they are less usable in clinical situations. GPs felt pressurised to prescribe, especially for fee-paying patients and in out of hours settings (OOH), suggesting the need for interventions that target the public’s perceptions of antibiotics. GPs behaviours surrounding prescribing antibiotics need to change in order to reduce AMR and change patients’ expectations.

**Electronic supplementary material:**

The online version of this article (10.1186/s12875-019-0917-8) contains supplementary material, which is available to authorized users.

## Background

Anti-microbial resistance (AMR) [1] is an increasingly serious threat to global public health [2, 3]. AMR is defined as the ability of pathogenic bacteria to withstand the actions of antibiotic drugs [4]. Increasing consumption of antibiotics is associated with the development of antibiotic resistance at individual, community, country and regional levels [5, 6]. It is recommended that the fewest number of effective antibiotic courses should be prescribed for the shortest period possible to minimise this effect [6]. A post-antibiotic era, in which common infections can kill, is a very real possibility for the twenty-first century [4]. Over the last 30 years, no major new types of antibiotics have been developed [4], therefore antibiotic stewardship programmes, dedicated to improving antibiotic use, have been established to conserve our diminishing antibiotic resource [7, 8].

Acute respiratory tract infection (ARTI), which incorporates the term “upper respiratory infection” (URTI), is the most common reason for antibiotic prescription in adults in the United Kingdom (UK) and Europe and these prescriptions are often inappropriate as they were prescribed to patients not necessarily needing an antibiotic [9, 10]. The benefits of antibiotics are marginal for the management of most cases of ARTI [11–13], including sore throat [14]. With few exceptions, unnecessary prescribing of antibiotics for patients with mainly URTI is common [15, 16]. Additionally, it is estimated that 75% of overall antibiotic prescribing takes place in primary care [17]. Antibiotic use in Ireland is considered to be mid-range in comparison to other European Union (EU) countries [18]. Thus explorative studies are required to understand GPs’ challenges in avoiding unnecessary prescription of antibiotics for ARTI. The most recent qualitative study in Ireland by Fleming et al. found that antibiotic prescribing is strongly influenced by the context of healthcare delivery and that the lack of implementation of guidelines and knowledge regarding antibiotic prescribing patterns are significant challenges that need to be addressed [19]. The aim of this paper, therefore, is to investigate why GPs in Ireland continue to prescribe antibiotics for ARTI, despite widely publicised guidelines and evidence of their ineffectiveness [20].

## Methods

### Study setting

Practices affiliated with the University of Limerick Medical School are based in three of Ireland’s four healthcare regions (Dublin Mid Leinster, South, West) and are broadly representative of general practices nationally by size, patient eligibility for free care and urban/rural location [21]. GPs from these locations work in urban, rural or mixed practices and experience ranged from less than ten years up to more than ten years. GPs work as independent contractors in the Irish primary care system. For patients to qualify for a medical card the applicant must earn below a certain figure based on family size, or be aged over 70 years or under 6 years. GPs (who are self-employed) are paid a ‘per capita’ fee by the state for their care. These patients do not pay directly for GP consultations whereas patients without a card pay an average of €50 per consultation. Patients who develop acute illness outside of normal consultation times (which are mostly between 9 am and 5 pm, Monday to Friday and all over weekends and bank holidays) are usually seen by an out of hours (OOH) service, which is manned by GPs. GPs working in OOH services do not have the same rapport with these patients as they would with their own patients. Private patients pay a fee for medical treatment; this fee can often be higher in an OOH setting. .

### Study design and participant recruitment

An explorative qualitative study design and purposive sampling was used for participant recruitment of 13 GPs [22]. This sampling method was used to ensure a broad spread of age and experience of the GP, as well as geographical location and level of establishment of their practice. All of the invited GPs agreed to participate in the study. Data analysis was carried out simultaneously by JOD and LFWL. In consultation with the research team JOD and LWFL came to the conclusion that data saturation has occurred because similar themes started to appear and chances of new themes to appear in subsequent interviews was judged to be minimal. Therefore no further participants were recruited. The majority of GPs have over 20 years’ experience in general practice and all the GPs worked in OOH setting. GPs were not offered any incentives to take part in this study. The characteristics of the participating GPs are shown in Table 1.

**Table 1:** Characteristics of GPs interviewed

Name/ID	Gender	Urban/Rural	Years in GP practice
GP 1	M	R	> 10
GP 2	M	R	> 10
GP 3	M	U	> 10
GP 4	M	R	> 10
GP 5	M	U	< 10
GP 6	F	U	< 10
GP 7	M	U	> 10
GP 8	F	U	> 10
GP 9	M	R	> 10
GP 10	F	R	> 10
GP 11	F	U	> 10
GP 12	M	U	< 10
GP 13	M	R	> 10

### Data collection

Individual semi-structured face-to-face interviews were conducted from June to August 2017 by a research assistant (LFWL) in the GP’s surgery during a break in their working day. The researcher was a trainee medical student at the time of data collection. The research assistant used an interview guide that was designed by two GP researchers (ROC, AOR), one social care researcher (JOD) and one public health researcher (SJP) (See Additional file 1: A). This guide was pilot tested with two GPs who had no involvement with this project to ensure clarity and global understanding of the terms used. The research assistant (LFWL) was provided with training to conduct semi-structured interviews. The researcher had no prior relationships with any of the interviewees. Each interview lasted on an average of 40 min and took place in a quiet room with only the interviewer and the interviewee present. Interviews began with a verbal explanation from the interviewer of the voluntary nature of the interview and the freedom to withdraw at any stage. The interviewees were given time to read the consent form and any resulting queries were answered by the interviewer. They were then given the opportunity to give their informed consent by signing the consent form (See Additional file 2: B).

### Analysis

Interviews were digitally recorded, transcribed and uploaded to NVivo (version 11). After each interview, the reviewers used thematic analysis as described by Braun and Clarke [23], to analyse the data gathered from the semi-structured interviews. The six phases in the analysis consist of “familiarizing yourself with your data, generating initial codes, searching for themes, reviewing themes, defining and naming themes and producing the report” [23]. The initial codes were developed by JOD, ROC and LFWL through reviewing the interviews and identifying similar codes among them. Following this, five of the researchers (SJP, AOR, LFWL, JOD, ROC) met to review and further analyse the codes. Similar codes were grouped together to develop themes. Themes were further reviewed by one author (CD) and all authors collaborated to define and name the themes and subthemes. Three authors then worked to produce the report (JOD, ROC, SJP). Throughout the analysis stage, codes and themes were defined, combined, refined and recoded in line with Braun and Clarke [23] (See Additional file 3: C). Supplementary Material C outlines the coding process. The guidelines for reporting of qualitative studies are outlined in Additional file 4: D. 

### Ethical considerations

Ethical approval for this study was granted by from the University Hospital Limerick Research Ethics Committee (Number: 068/17).

## Results

In total, 13 GPs were interviewed as part of this study. The characteristics of each participating GP are summarised in Table 1. We identified three main themes from the emerging data; (1) non-comprehensive guidelines; how guideline adherence can be difficult, (2) GPs under pressure; pressures to prescribe from patients and perceived patient expectations and (3) Unnecessary prescribing: how to address it and the potential of public interventions to reduce it.

### Non-comprehensive guidelines

GPs interviewed were aware that antibiotics are being used inappropriately for non-bacterial minor illness such as ARTIs. GPs believed that this can lead to AMR. The GPs were also aware that over the last thirty years, there has been no new class of antibiotic introduced and that AMR is a real public health threat. GPs acknowledged that there are guidelines on antibiotic prescribing but explained that there can be challenges when trying to decide on prescribing antibiotics, especially when neither the person’s condition is clear-cut nor the length of time that they have been having symptoms. Many factors must be taken into account before a decision is made on prescribing antibiotics such as age, general health or any other pre-existing health conditions of the patient. According to the participants, the existing guidelines outlined by the Health Service Executive for ARTIs are not comprehensive, and do not clearly outline for a multitude of factors such as cough, sinus pain and the best course of action for all conditions the GPs face during their consultations.

*“The lines are blurred when it comes to people with COPD and bad chest infections. My threshold is lower with giving them an antibiotic” (GP 1).*

*“There was a study done when I was in paeds training – GP’s were not prescribing early enough in chest infections in children and they ended up being admitted with pneumonia because they weren’t getting antibiotics” (GP 6).*

### GPs under pressure

In this second theme, GPs discussed how prescribing patterns for antibiotic management of ARTI are influenced by many factors.

#### Private versus free health care patient

One factor mentioned by the GP was the cost factor. GPs mentioned how prescribing pattern might be influenced depending on whether the patient is paying or has access to free healthcare. This is another ethical challenge which GPs face in OOH settings; they indicated that some GPs find it difficult to let a private patient leave the consultation without a prescription for an antibiotic because they are paying a fee. GPs are trying to satisfy the perceived patients’ expectations for antibiotics. It appears that the private health service model presents a potential conflict for the GP who has a duty to provide healthcare based on best evidence but must also satisfy private patients’ expectations in order to retain them. Underlying this subtheme is the concept that GPs are trying to satisfy the perceived patients’ expectations for antibiotics.

*“Sometimes with private patients they feel that because they are paying you a fee, they should be getting a prescription and that it should be a prescription for as they call it "a strong antibiotic” (GP 3).*

*“Thankfully I work with mostly medical card patient who are less demanding than private patients. Private patients expect to get an antibiotic...can you imagine trying to send a private patient home with advice on Paracetamol rest and fluid for €50. A lot of them wouldn’t be pleased” (GP 6).*

#### Perceived patient expectations

A third factor, influencing the GPs to prescribe antibiotics was patient expectation. Two of the GPs indicated that patient expectations were often influenced by past experience. These patients had a pattern of attending for certain conditions and expected to be treated with antibiotics rather than having a conversation about the best treatment pathway. GPs commented on how patients often had high expectations of receiving an antibiotic. GPs felt that this high level of expectation was borne out of a pattern of behaviour, which developed when receiving antibiotics for ARTI in the past. There is a big issue with this subgroup of the population who are reliant on receiving antibiotics and who hold the belief that they will not be cured without one.. When GPs think that patients have learned behaviours, for example when they have received antibiotics in the past for trivial upper respiratory symptoms and expect to do so again now, this can affect how they manage a patient. Their own clinical awareness may be undermined by patients’ expectations thus resulting in GPs prescribing unnecessary antibiotics.

*“So if you were brought along as a young fella and you sniffled then you go along as an adult most of the time when you sniffle. If the opposite pertained and you were only brought to the doctor occasionally when people actually thought there was something wrong with you well then you tend to hold back a bit and only go when you need to go so a lot of this is learned behaviour” (GP 5).*

*“Then you have another cohort and all they want is an antibiotic and that is what they are used to getting and they are a lot more challenging” (GP 8).*

Limited duration of consultations was a major factor. Treating patients in OOH settings are different to usual consultations; the GP may have no previous relationship with the patient or knowledge of the patient’s history so it can be difficult to know how a patient has been treated for a similar presentation in the past. Also it can be difficult to elicit the patient’s views on the benefits of antibiotics and whether the main purpose of their visit is for medical assessment of their illness and symptomatic treatment only if that is all that is indicated. This raises an ethical challenge; how do GPs maintain professionalism while treating patients in busy OOH settings. GPs should carefully consider how to deal with patients during a consultation in OOH with limited time. GPs were also cognisant of the fact that it can be difficult to have the discussion with patients around their actual need for antibiotics. They indicated that they sometimes concede to patients´ demand for antibiotics and prescribe antibiotics even though they know it will not solve the patients’ condition and may contribute to AMR.

*“If they really want it I often end up giving the antibiotic but I’ll say – ‘I don’t think it will do you any good, it may have more side effects’. Out of hours it’s not even worth having that discussion, if they are in that pattern of behaviour… you are not going to change it” (GP 2).*

### Unnecessary prescribing: How to address it?

GPs discussed how they were aware that much of the prescribing of antibiotics for ARTIs in practice is unnecessary. They suggest the need for different interventions that target GPs which will help to prevent over-prescribing. GPs indicated that they use delayed prescriptions as a strategy to prevent the misuse/overuse of antibiotics. A delayed prescription is a valid prescription that a patient is given at the end of a consultation with directions to not use it unless they are still feeling unwell sometime later. One of the main reasons that GPs provided patients with delayed prescriptions was they suspected the patient’s clinical condition may decline in the coming days resulting in the need for a prescription. Doubt amongst GPs on the capacity for self-care by patients is another factor in issuing them.

*“I think sometimes the compromise there is the deferred script but you say ‘I don’t think you need to go on something right away. Hold off, there is a prescription for [name of an antibiotic], three times daily for 5 days but I would be hoping you don’t need to fill it’” (GP 3).*

*“But if it is an upper respiratory that probably looks viral but could deteriorate but coming up towards the end of the week, I would probably give them a deferred script” (GP 13).*

One GP made the argument for software to be used to help GPs track their prescribing habits. Through doing this, it could encourage them to be conscious of what drugs and how many they are prescribing to patients. According to the GPs, if they had something that would enable them to become more aware of their prescribing habits, they may be less inclined to prescribe antibiotics. Another GP believed that there should be penalties for GPs who are over-prescribing and this may encourage them to become aware of their prescribing habits in the future. The justification behind the penalties was that GPs might be lacking in knowledge in regards how much they are prescribing and even if they had management software to track their prescribing patterns, they may still not adhere unless they were penalised.

*“If there was something in your practice management system that you could switch on that tracked your prescribing habits and gave an automatic read out every month in relation to what you prescribed and then compared it” (GP 7).*

*“I think maybe if GPs were penalized for using antibiotics that might work. I can’t see...I know there are very good advertisements but they don’t seem to work. People have this idea “oh that applies to somebody else but not to me. Mine is a deserving case”. So they don’t identify with that person. So I think in some way to penalize GPs in some way for using antibiotics would be the way forward” (GP 10).*

Another suggestion by the GPs was to develop a system where antibiotics are prescribed based on a decision making process involving two medical professionals (GP and nurse) consulting with one patient. In the majority of cases, this would help to reassure the patient that they do not need antibiotics and help GPs to prescribe antibiotics based on clinical symptoms and not influenced by the factor mentioned in the previous theme.

*“Just watching my own nurses. If they say it to them, you know, I don’t…“we’ll see what the doctor says but I don’t think he needs the antibiotic” then you are beginning to push an open door now as distinct from starting from scratch and I think there might be a role for that” (GP 5).*

#### Intervention targeting the public

The GPs also suggested interventions that could be used in targeting the public and their perception of antibiotics. One GP mentioned how if children were taught in schools about antibiotics, this might be beneficial to the children and their parents. This GP also discussed how children are becoming more educated, through the medium of children’s television programmes, about what happens when they visit a doctor. They learn what to expect at visits in terms of how the GP will check to see if they are feeling unwell, and how the GP will make his/her decision on whether or not they need medicine to make them better. One participant mentioned that children have brought in a ‘Doc McStuffins’ doll so they are becoming more aware of what they do in a consultation.

*“School would be very good because children would come home with whatever their teacher said so if they had it in a book in school or a little video” (GP 13).*

*“A lot of patients coming in with Doc McStuffin dolls and at least we can check their ears and throats now. Yeah so little things like that would be good” (GP 13).*

## Discussion

### Main findings

This paper specifically investigates what factors are involved in the decision behind prescribing antibiotics for ARTI symptoms, despite GPs knowing the dangers and issues about antimicrobial resistance. GPs acknowledged that it is difficult to consistently implement existing guidelines because, according to them, guidelines tend to look at single symptoms in a unidimensional way while in real life consultations are more complex which makes the guidelines less usable. They also discussed how they felt pressurised to prescribe, especially for fee-paying patients. GPs discussed how they were aware that much of the prescribing of antibiotics for ARTIs in practice is unnecessary. They suggested the need for different interventions that target GPs and the public. Balancing the pressure to prescribe for patients diagnosed with an ARTI and increasing antimicrobial resistance is a key concern for the GPs currently.

Factors such as local resistance patterns, diagnostic uncertainty and increased patient demand often make it difficult to apply guidelines to everyday clinical situations, thus simply publishing guidelines is unhelpful [24]. Multifaceted educational interventions in general practice have been shown to be effective in reducing antibiotic prescribing [25, 26]. Guidelines must also be applicable to everyday clinical practice and meaningful in the local context [27]. GPs may need further guidance on how to address the concerns of patients without interpreting these questions as a demand for antibiotics [28]. However, adhering to prescribing guidelines can be hindered by the fact that they vary considerably regarding consistency of grading of the quality of evidence and strength of recommendations. Also as indicated by the GPs, guidelines do not take into consideration the variety of issues that may come up during a consultation [29].

While interviewees felt a certain pressure to prescribe antibiotics for public patients who were seen free of charge, there was a much stronger pressure felt to prescribe antibiotics for those patients who were paying the doctor. This commercial aspect of clinical life can affect the decision making of a GP; if they don’t prescribe for a private patient they could potentially upset them and lose them, thus reducing income and making their practice less commercially viable. This conflict between the doctor’s ethical duty and commercial reality needs to be taken into account in formulating guidelines and in changing the public mind set. This is probably best done by multifaceted education which has a strong public element so that patients are aware of the harms of unnecessary antibiotic prescribing [30]. This public educational policy should also have the effect of changing learned behaviour and helping patients realise that it is not necessary to treat all respiratory infections with antibiotics, even if this was their experience in the past. The centrality of the clinical decision can be undermined or overshadowed by subtle contextual factors such as payment fostering the desire to “give the paying patient something tangible”.

Patterns of antibiotic prescribing that clearly do not adhere to guidelines have been reported in the OOH setting [31, 32]. However, the higher OOH prescribing rates could be at least partly explained by a different population of presenting patients [33]. In Norway, antibiotic prescribing for ARTIs in OOH services was at a similar level to that of normal working hours [34]. It has been suggested that this is because doctors working in OOH units are more adherent to guidelines than doctors working in regular general practice, although rates of antibiotic prescribing increased during busy sessions [34]. It must be acknowledged therefore that the OOH clinical setting is a different and the usual rules may not apply. Guidelines and interventions to reduce antibiotic prescribing should reflect this.

The published literature indicates that patients with a lower education level or who come from more deprived socioeconomic backgrounds are more likely to be prescribed antibiotics for ARTI [35–37]. However, studies from Ireland, China and Malaysia have shown that patients, often having better socio economic conditions, paying a fee to the healthcare professional are also more likely to receive antibiotics for ARTI [38–40]. The feeling among many interviewees was that not prescribing an antibiotic was tantamount to letting the patient “walk away empty handed”, which might lead to low patient satisfaction resulting patients changing their GP. There is the ethical question for GPs; should they let the pressure to prescribe for private patients overshadow their own professionalism and GPs find it difficult to let a private patient leave the consultation without a prescription for an antibiotic because they are paying a fee. In 2016, the Irish Medical Council published their eighth edition of its guide to professional conduct and ethics which describes professionalism [41]. It recommends how to incorporate the core components of professionalism into best practice by putting the interests and wellbeing of the patient first and teaching GPs how to deal with conflicts of interest [41]. However, similar to the clinical guidelines, this guide also seems to have been of limited help to the GPs to manage their challenges. The aforementioned feeling among GPs was that, because the consultation allows the GP to form an evidence-based opinion as to the non-bacterial nature of the disease, their role is in educating the patient and non-antibiotic treatment of symptoms such as pain and fever. It is true that patient satisfaction varies with antibiotic prescription policies for ARTI and patients are less satisfied in practices with low antibiotic prescribing rates [38] or when they expect but are not prescribed antibiotics [42, 43]. A strategy of delayed prescriptions was commonly employed by interviewees. There is evidence that the use of delayed prescriptions has been associated with reduced antibiotic use [42, 44]. In a qualitative study by Edelstein et al., they argue that due to the pressure from patients to obtain antibiotics, GPs are prescribing them ‘just in case’ due to fear of litigation or patient deterioration [45]. However, delayed prescriptions may be useful in allowing the GP to give focused education to the patient about the expected natural history of their ARTI and what symptoms and signs to look out for that might indicate deterioration. Patient focused education combined with the use of educational leaflets or booklets has been shown to reduce antibiotic consumption in children and adults [46, 47]. Mass media campaigns have also been shown to work to reduce prescribing of antibiotics [48]. Additionally training for GPs to improve their communication skills is also an important aspect to focus on. This will help the GPs educate patients, however limited the scope might be, on how ineffective antibiotics could be for their condition and the harmful effects of unnecessary use of antibiotics [49, 50]. However, limited consultation time might pose challenge for GPs to educate the patients. Use of nurse practitioners might be a feasible solution to be considered here. Williams et al’s recent study suggested that the use of nurse practitioners may encourage more consistent prescribing patterns [51]. Similarly, Rowbotham et al’s study also suggests that nurse practitioners will educate patients and address patients concerns rather than prescribe antibiotics [52].

GPs believed that strategies such as practice management software and penalties for over-prescribing could be potential tools to reduce antibiotic prescribing and the development of antimicrobial resistance. One study investigated whether a clinical support system embedded into electronic health records could reduce antibiotic prescribing for ARTI in primary care [53]. The results did show a slight decline in prescribing rates. Liao et al. recently studied the issue of financial penalties for inappropriate antibiotic prescribing for ARTI among practicing American physicians [54]. In a randomised web based trial using clinical vignettes, they showed that support for financial penalties targeting inappropriate antibiotic prescribing was highest among physicians who received information about patient harms [54]. This also highlight the importance of educating GPs on the harmful effect of unnecessary antibiotic prescription for patients, not about AMR only. GPs stated that they do give into patients and prescribe antibiotics even though they know it will not solve the patients’ condition and may contribute to AMR. GPs need training in communication skills to talk to the patients and not to “give up” too easily [55]. GPs outlined that limited durations of consultations were a factor in their decision to prescribe antibiotics. Training GPs to communicate more effectively and efficiently with their patients may enable them to be more confident, change their prescribing behaviours and reduce antibiotic prescribing. One study [56] showed internet based training on enhanced communication skills lowered antibiotic prescribing rates.

In our study, one GP suggested that schools would be a good place for children to get more education on antibiotics and AMR, which may be beneficial to them. Research shows that primary health care teams which include nurse practitioners can improve patient follow-up and disease management by advancing effectual health promotion and disease prevention [56]. The use of nurse practitioners or practice nurses working in conjunction with the GP in the management of minor illness such as ARTI should be tested as an option for enhancing guideline implementation. In addition to measures to control the prescription rates, efforts focused on complete prevention of unnecessary prescriptions should also be initiated, preferably starting during medical school training. A study in Portugal of high school students who were involved with a programme to promote awareness of antibiotic resistance, showed that students developed a more comprehensive picture of antibiotic resistance [57]. It is interesting to note that the strategies suggested by GPs participating in our study was mainly to control, but not to prevent, unnecessary and increased antibiotic prescription. The results of this should inform further guideline development and implementation, especially taking into account the reasons given by healthcare providers for not consistently using antibiotic guidelines in the management of ARTI.

### Strengths and limitations

There have been very few studies exploring the views of GPs on AMR and prescribing of antibiotics for ARTI in an Irish context. This study adds to the existing literature in the area of prescribing antibiotics for ARTI. It also compliments existing papers on AMR and prescribing of antibiotics for ARTI. GPs’ extensive experience in general practice has also given us insights into how antibiotic prescribing has changed over time. There is potential selection bias since the authors and the participants were affiliated to the same institution. However, effect of such bias might be limited since studies from other settings suggest similar findings [48]. However, in order to reduce the risk of such bias, future work could include a larger sample of GPs to be interviewed. GPs who did not take part in this study may have different views about prescribing antibiotics, the usefulness of guidelines in trying to reduce AMR and whether perceived patient expectation influences their prescribing of antibiotics.

## Conclusions

More comprehensive guidelines aimed at reducing antibiotic prescribing rates for ARTI, should involve prior collaboration between policy makers and GPs. Such guidelines must take into account contextual considerations such as: reducing patient expectation by education and increasing awareness of AMR; developing policies for prescribing for fee-paying patients and acknowledging the perceived pressure to prescribe in busy OOH settings. GPs should also be more aware how to change learned behaviours when prescribing antibiotics for patients with ARTI. GPs should also be more aware how to change learned behaviours when prescribing antibiotics for patients with ARTI.

## Additional files

Additional file 1: A: Interview Guide. Interview Guide used in each the thirteen semi-structured interviews. (DOCX 18 kb)

Additional file 2: B: Consent Form. Consent form which was interviewee reviewed and signed prior to being interviewed. (DOCX 20 kb)

Additional file 3: C: Coding Process. A table with three columns outlining the coding process of each interview. (DOCX 15 kb)

Additional file 4: D: COREQ Guidelines. Guidelines used for reporting qualitative studies. (PDF 133 kb)

## Abbreviations

<: Less than
>: Greater than
AMR: Antimicrobial Resistance
ARTI: Acute Respiratory Tract Infection
ARTIs: Acute Respiratory Tract Infections
DDD: Defined Daily Doses
EU: European Union
F: Female
GPs: General Practitioners
M: Male
OOH: Out of Hours
R: Rural
U: Urban
UK: United Kingdom
URTI: Upper Respiratory Infection
