# Supplementary material for: Over prescribing of antibiotics for acute respiratory tract infections; a qualitative study to explore Irish general practitioners’ perspectives
Source: BMC Fam Pract. 2019 Feb 14;20:27. doi: 10.1186/s12875-019-0917-8 (PMC6374900; doi:10.1186/s12875-019-0917-8)
Supplement: Supplementary file 1 — A: Interview Guide. Interview Guide used in each the thirteen semi-structured interviews. (DOCX 18 kb) [file 12875_2019_917_MOESM1_ESM.docx]

**Supplementary Material A: Interview Guide**

1. Introduce the interviewer and the research project. Repeat the information on the information leaflet and emphasize the voluntary nature of their participation.

2. Could you please tell me a little bit about yourself? (how long you have been working as a GP, how long at this OOH service, year of qualification, part time/full time GP.)

3. What are your views on antibiotic use in Ireland?

4. What are your views on prescribing antibiotics?

5. How often do you consult patients presenting with upper respiratory (URTI) and how do you treat them?

6. According to you, what is most commonly expected disease management strategy by the patients?

7. What are your views on the use of antibiotics to treat upper respiratory (URTI) conditions presenting acutely in GP?

8. How often do you prescribe antibiotics to treat URTI?

9a. Could you please describe a scenario where you would prescribe antibiotics to treat URTI?

9b. Probing question? how would you be influenced by duration of the cough, presence of fever, presence of green nasal discharge, presence of physical signs such as tonsillar exudate, red tympanic membrane, creps on auscultation

9c. Other than patient symptoms what else would influence you in prescribing or not prescribing an antibiotic.

10. Similarly, under what circumstances would you not prescribe antibiotics?

11. Can you think of any situation where you would have changed your antibiotic prescription e.g. to a different antibiotic or no antibiotic?

12a. Would your treatment strategy or views on antibiotic prescription change when seeing the patient in an out of hours setting?

12b. If yes, please explain.

13a. Would the out of hours patient profile be different than a routine consultation?

13b. If yes, please explain.

14a. Would the patient being private have a bearing on your decision to Rx an antibiotic

14b If yes, please explain

15a. What are your views on the expectations from patients for the

15b. Prescription of antibiotics to treat URTI presenting acutely in GP?

15c. Non antibiotic management of URTI presenting acutely in GP?

16. How do you deal with a patient who insists on antibiotics despite your recommendations?

17. How do pharmaceutical reps introduce new drugs to the GP. Does it influence your prescribing pattern and how?

18. Do you need more guidelines and support? Are there specific types of training you recommend?

19. Are there any issues of relevance to antibiotic prescription and URTI, which I have failed to ask?
