# Supplementary material for: Over prescribing of antibiotics for acute respiratory tract infections; a qualitative study to explore Irish general practitioners’ perspectives
Source: BMC Fam Pract. 2019 Feb 14;20:27. doi: 10.1186/s12875-019-0917-8 (PMC6374900; doi:10.1186/s12875-019-0917-8)
Supplement: Supplementary file 2 — B: Consent Form. Consent form which was interviewee reviewed and signed prior to being interviewed. (DOCX 20 kb) [file 12875_2019_917_MOESM2_ESM.docx]

**Supplementary Material B: Consent Form**

**Title of Study:** **A study on patients’ and GP’s attitudes to the prescribing of antibiotics for acute upper respiratory infections.**

| *I have read and understood the* ***Information Leaflet*** *about this research project. I have been able to ask questions, which have been answered to my satisfaction.* | ***Yes*** | ***No*** |
| --- | --- | --- |
| *I understand that my participation in the study is voluntary and that I can opt out at any time without giving any reason.* | ***Yes*** | ***No*** |
| *I am aware of the potential risks of this research study.* | ***Yes*** | ***No*** |
| *I have been given a copy of the Information Leaflet and a completed consent form for my records.* | ***Yes*** | ***No*** |
| ***Storage and future use of information:***  *I give my permission for information collected about me to be stored or electronically processed for the purpose of scientific research and to be used in related studies or other studies in the future but only if the research is approved by a Research Ethics Committee.* | ***Yes*** | ***No*** |

*Participant Name (Block Capitals): __________________________*

*Participant Signature: _____________________________________*

*Date: ___________________*

***To be completed by the Principal Investigator or his nominee.***

*I the undersigned, have taken the time to fully explain to the above patient the nature and purpose of this study in a manner that they could understand. I have explained the risks involved as well as the possible benefits. I have invited them to ask questions on any aspect of the study that concerned them.*

*Name (Block Capitals): _____________________________________*

*Qualifications: _______________________________*

*Signature: _______________________________________________*

*Date: ________________*

*3 copies to be made: 1 for patient, 1 for PI and 1 for practice records (if relevant).*
