# Supplementary material for: Over prescribing of antibiotics for acute respiratory tract infections; a qualitative study to explore Irish general practitioners’ perspectives
Source: BMC Fam Pract. 2019 Feb 14;20:27. doi: 10.1186/s12875-019-0917-8 (PMC6374900; doi:10.1186/s12875-019-0917-8)
Supplement: Supplementary file 3 — C: Coding Process. A table with three columns outlining the coding process of each interview. (DOCX 15 kb) [file 12875_2019_917_MOESM3_ESM.docx]

**Supplementary Material C: Coding Process**

| **Interview Quotation** | **Coded as** | **Subtheme** |
| --- | --- | --- |
| “We would see an awful lot more children under the age of six who’s parents are looking for treatment and more specifically they are looking for an antibiotic. I would see an awful lot more children whose parents or carers request an antibiotic”. | Requesting antibiotics | Patient expectations |
| “But the expectation for antibiotics is higher when people have gone to the bother of making an appointment with [Out of hours service] and you think maybe an antibiotic is not going to help here, it’s often difficult because of the pattern and because they have gone through that”. | Expectation for antibiotics | Patient expectations |
| “I think sometimes the expectations with public patients is that they would always get an antibiotic. Sometimes with private patients they feel that because they are paying you a fee that they should be getting a prescription and that it should be a prescription for as they call it “a strong antibiotic”. | Paying versus not paying for antibiotics | Patient expectations |
| “Like any patient can come back but a public patient can come back more easily, lets say. Private patients come in. They are under pressure. They are out of work, off work or they have been sent home from work and I totally understand”. | Private patients consultations | Patient expectations |
